# The *BRCA1* c.4096+1G>A Is a Founder Variant Which Originated in Ancient Times

**DOI:** 10.3390/ijms242115507

**Published:** 2023-10-24

**Authors:** Paolo Aretini, Silvano Presciuttini, Aldo Pastore, Alvaro Galli, Sara Panepinto, Mariella Tancredi, Matteo Ghilli, Chiara Guglielmi, Diletta Sidoti, Caterina Congregati, Maria Adelaide Caligo

**Affiliations:** 1 Fondazione Pisana per la Scienza, San Giuliano Terme, 56017 Pisa, Italy; aldo.pastore@sns.it; 2 Dipartimento di Ricerca Traslazionale e Nuove Tecnologie in Medicina e Chirurgia, Università di Pisa, 56126 Pisa, Italy; silvano.presciuttini@gmail.com; 3 Laboratorio NEST, Scuola Normale Superiore, 56126 Pisa, Italy; 4 Istituto di Fisiologia Clinica, Consiglio Nazionale delle Ricerche (CNR), 56124 Pisa, Italy; alvaro.galli@cnr.it; 5 Laboratorio di Genetica Molecolare, Azienda Ospedaliera Universitaria Pisana, 56126 Pisa, Italy; sarapanepinto92@gmail.com (S.P.); mariella.tancredi@ao-pisa.toscana.it (M.T.); chiara.guglielmi.cg@gmail.com (C.G.); diletta.sidoti.5@gmail.com (D.S.); 6 Breast Unit, Azienda Ospedaliera Universitaria Pisana, 56126 Pisa, Italy; m.ghilli@ao-pisa.toscana.it; 7 Genetica Medica, Azienda Ospedaliera Universitaria Pisana, 56126 Pisa, Italy; c.congregati@ao-pisa.toscana.it

**Keywords:** hereditary breast and ovarian cancer, *BRCA1*, pathogenic variant, founder variant, Italy

## Abstract

Approximately 30–50% of hereditary breast and ovarian cancer (HBOC) is due to the presence of germline pathogenic variants in the *BRCA1* (OMIM 113705) and *BRCA2* (OMIM 600185) onco-suppressor genes, which are involved in DNA damage response. Women who carry pathogenic *BRCA1* variants are particularly likely to develop breast cancer (BC) and ovarian cancer (OC), with a 45–79 percent and 39–48 percent chance, respectively. The *BRCA1* c.4096+1G>A variant has been frequently ascertained in Tuscany, Italy, and it has also been detected in other Italian regions and other countries. Its pathogenetic status has been repeatedly changed from a variant of uncertain significance, to pathogenic, to likely pathogenic. In our study, 48 subjects (38 of whom are carriers) from 27 families were genotyped with the Illumina OncoArray Infinium platform (533,531 SNPs); a 20 Mb region (24.6 cM) around *BRCA1*, including 4130 SNPs (21 inside *BRCA1*) was selected for haplotype analysis. We used a phylogenetic method to estimate the time to the most recent common ancestor (MRCA) of *BRCA1* c.4096+1G>A founder pathogenic variant. This analysis suggests that the MRCA lived about 155 generations ago—around 3000 years ago.

## 1. Introduction

About 10–30% of BCs and OCs show familial clustering, but only 5–10% of cases are estimated to be hereditary, being associated with a germline pathogenic variant (PV) or likely-pathogenic variant (LPV) in a cancer predisposition gene [1]. A single-center study of 488 breast cancer patients found a *BRCA1*/*2* pathogenetic variant in 6.1% of women (5.1% in non-Ashkenazi Jewish patients) [2]. Another study found that 2.5% of unselected breast cancer patients carry pathogenic *BRCA1*/*2* variants [3]. Of those diagnosed with ovarian cancer, 15% were found to harbor germline mutations in *BRCA1* or *BRCA2* [4]. *BRCA1* (OMIM 113705) and *BRCA2* (OMIM 600185) are tumor suppressor genes, which are involved in DNA damage response [5,6].

Women who carry pathogenic *BRCA1* variants are particularly likely to develop breast cancer (BC) and ovarian cancer (OC), with a 45–79% and 39–48% chance, respectively [7,8,9,10,11].

In general, *BRCA1* and *BRCA2* exhibit broad mutation spectra across populations; however, specific pathogenic variants reach high relative frequency in some ethnic groups, such as Ashkenazi Jews or Icelanders [12,13].

In the Italian population, some mutations have a higher frequency, and there are instances of founder mutations. For example, the *BRCA1* pathogenic variant c.5083del19 was detected in 24 patients with BC and/or OC belonging to 24 unrelated families from Calabria [14]. Among these patients, haplotype analysis identified a common allele associated with a possible common ancestor. Several studies have shown that the BRCA1 5083del19 mutation is also present with a high frequency in the Sicilian population [15,16,17]. These studies have shown the presence of a common allele in the families analyzed, thus suggesting the presence of a founder effect in the Sicilian population. Another founder mutation, *BRCA1* 1499insA (c.1380dup), was discovered by Marroni et al. in the Tuscany population. This mutation was present in an ancestor dating back 750 years [18].

In regard to the methods to date the pathogenic variants, during recent years, several methods have been developed [19,20]. The most common methods for dating variants fall into three broad categories: methods based on pathogenic variant frequencies, methods based on gene trees, and methods based on linkage disequilibrium decay [21,22,23]. However, these methods have limitations. This is particularly true when the sample size is small and when high density SNPs data are used. For this reason, a more recent phylogenetic method based on the haplotype sharing approach is used. It consists of three steps: (1) determining the phase of a dense microsatellite marker map around the gene of interest in collected families; (2) determining the length of the haplotype shared by all pairs of variant carriers in the sample and arranging the length in a triangular similarity matrix; and (3) converting the similarity matrix into a distance matrix, from which the evolutionary tree of the studied pathogenic variant can be inferred. Using this method, Marroni et al. estimated that the common ancestor of *BRCA1* 1499insA (c.1380dup) pathogenic variant carriers lived in Tuscany, Italy, and that this variant was already present in the population of the late Middle Ages (about 30 generations ago) [18].

The variant c.4096+1G>A, also known as IVS11+1g>a, was identified in a 52-year-old woman with hereditary breast and ovarian cancer (HBOC) in Tuscany, Italy [24]. It was also reported—albeit sporadically—in other Italian regions [25,26], and in other countries such as Brazil, Greece, Turkey, and the United States [27,28,29,30]. The variant removes the donor splice site of exon 11, the longest exon of *BRCA1* (3426 bp), encoding protein signals necessary for nuclear localization. Regulation of the splicing process involving exon 11 results in three main transcriptional products: (1) the full-length isoform; (2) the ∆11 isoform (skipping of exon 11), which results in a protein with an in-frame deletion of 1142 amino acids; and (3) the ∆11q isoform (partial exon 11 skipping), resulting in a protein with an in-frame deletion of 1103 amino acids (p.Ser264_Gly1366del) due to the use of an alternative splice donor site located 117 bp downstream at the beginning of exon 11 [31,32]. The functional significance and the role of these alternative splicing products in tumorigenesis processes is not fully understood yet [33,34]. This variant has been reclassified several times over the years, and the interpretation of this variant is conflicting even today; however, the variant can be classified as pathogenic according to the original ACMG/AMP (American College of Medical Genetics/Association of Molecular Pathologist) criteria: PVS1 very strong, PM2 supporting, and PS1 moderate. Using instead the recently revised ClinGen ENIGMA BRCA1 and BRCA2 Expert Panel Specifications criteria, the variant can be re-classified as likely pathogenic (PVS1 moderate, PM2 supporting, PS1 moderate, and PP1 supporting) [35]. In addition, on Varsome (https://varsome.com/variant/hg19/BRCA1%3Ac.4096%2B1G%3EA?; accessed on 1 October 2023) or on BRCAexchange (https://brcaexchange.org/variant/873890; accessed on 1 October 2023), the variant is classified either as pathogenic-likely, pathogenic, or VUS. In the LOVD database, the variant is classified as pathogenic (https://databases.lovd.nl/shared/genes/BRCA1; accessed on 1 October 2023). However, the frequency of c.4096+1G>A variant in our cohort of BRCA1 carriers probands is 10% (38 out of 364). Whereas, the frequency of the BRCA1 c.4096+1G>A variant on total number of individuals screened because of a personal and/or familial breast/ovarian cancer history is 0.97% (38/3803).

In this study, we determined the most recent common ancestor (MRCA) of the *BRCA1* c.4096+1G>A variant in 27 probands from 27 independent Tuscan families with a history of BC and/or OC. The DNA was typed with Illumina Infinium OncoArray, and the gametic phase was inferred by free available software. Finally, we dated the variant c.4096+1G>A using a modified Marroni et al. phylogenetic method [18]. This analysis suggests that the MRCA lived about 155 generations ago—around 3000 years ago. We confirm these results by using the method of Gandolfo et al. [35].

## 2. Results

### 2.1. Haplotype Analysis

The diplotypes inferred by the phasing software for the 27 supposedly independent carriers were paired with each other (351 pairs) to determine the length of the carrying haplotype shared by each pair. Although this task can be accomplished via software, we preferred to visually inspect the BRCA1 region, looking for possible false recombinations or rare typing errors. In fact, a preliminary analysis of a trio (parents with a child) had shown an excess amount of imputed recombinations compared with those expected given the genetic length of the investigated segment.

**Figure 1:**
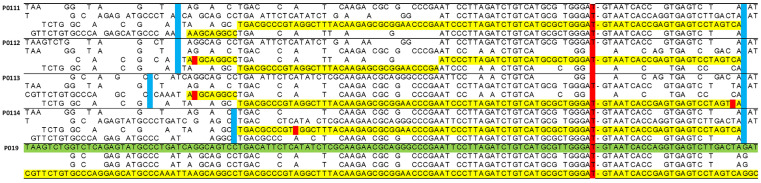
Small snippet of the spreadsheet used to determine the length of the carrier haplotype shared by pairs of individuals. The analyzed segment is delimited by markers chr17_39890876_C_T (position 39,890,876, **left**) and rs12948043 (position 41,615,830, **right**), thus encompassing 1.7 Mb or 1.2 cM. The implemented algorithm requires four lines for each subject to decode the two strings of 0 and 1 provided by the phasing software into the original DNA bases. Here, the reference subject is P019 (**bottom**), whose two inferred haplotypes are highlighted in yellow (carrier) and green (non-carrier). The yellow lines of the tested subjects (here P0111, P0112, P0113, and P114) highlight their carrier haplotype shared with P019. The blue positions, left blank by the algorithm, indicate markers where test and reference subjects are homozygous for alternative alleles; these represent “hard stops”; i.e., markers where any shared haplotype is necessarily broken off. Red dots mark suspicious typings (which are practically irrelevant here). In subject P012, the phasing software proposes what is to be interpreted as a spurious double recombinant. Eliminating these unreliable “soft” breaks leads to a more conservative estimate of the time to the MRCA.

Figure 1 shows a small fragment of the spreadsheet used for this task, where the diplotype of subject P019, who shares by far the shortest haplotype with all others, is compared to four other subjects. A vertical red bar highlights a marker internal to BRCA1.

The triangular matrix with the extremes in Mb of the haplotype shared by each pair was then converted into a triangular matrix showing the extremes in cM.

**Figure 2:**
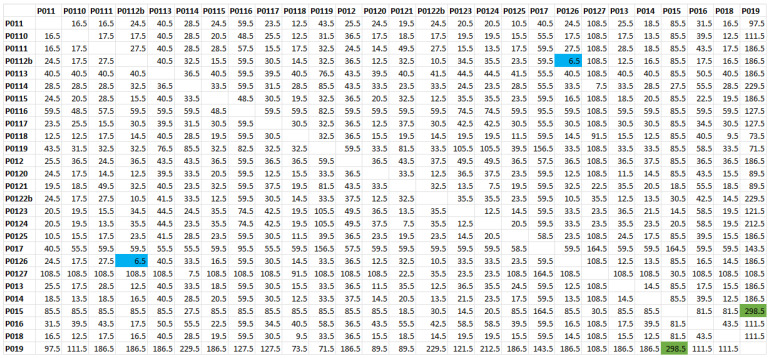
Symmetric square matrix of the estimated number of generations separating each pair of individuals from their MRCA carrying the c.4096+1G>A variant. Blue: minimum value (the common ancestor of P0126 and P0112b lived 6/7 generations ago). Green: maximum value (about 300 generations from the common ancestor of 019 and P015). The randomness of the recombination process that shortens the haplotype shared by multiple individuals descending from the same MRCA in different ways is taken into account by the tree-building algorithm.

The difference in cM between the positions of the extremes of the haplotype shared by each pair of individuals, representing the segment length, was then computed. The number of generations based on the simulation applied to the distance matrix in cM is shown in Figure 2. The generated matrix is first converted into generations, and then into the number of meioses separating each pair of individuals (Figure 2). The maximum length (20.85 cM) was obtained for the pair P0126-P0112b. This segment encompasses almost the entire chromosomal region analyzed; it indicates a recent common ancestor between these two individuals, living approximately six or seven generations ago (blue boxes, Figure 2). Otherwise, the shortest feature length (0.42 cM) was observed between P019–P015, indicating a distant common ancestor living 300 generations ago (green boxes, Figure 2).

### 2.2. The Time to the MRCA

**Figure 3:**
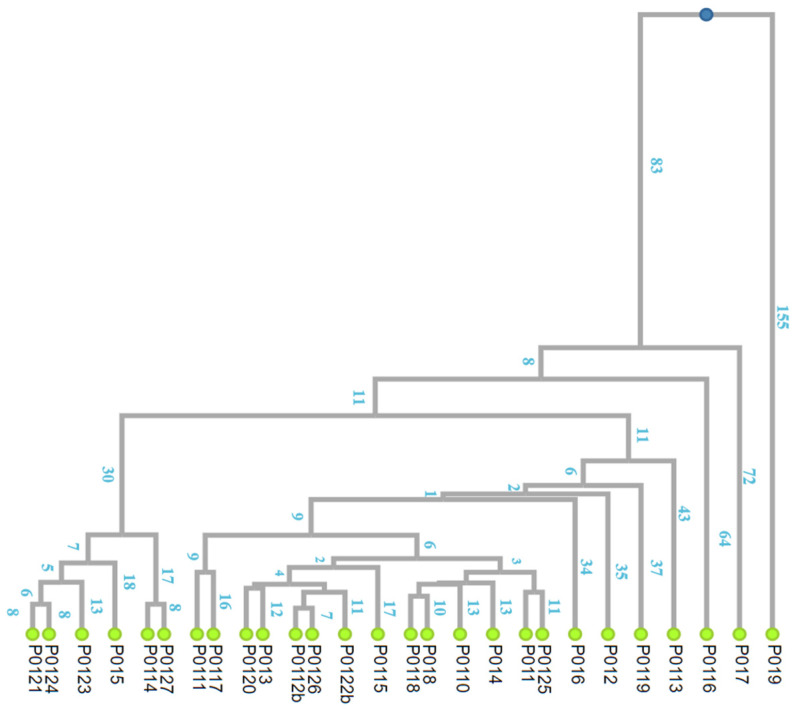
Extended genealogical tree for 27 independently families carrying the BRCA1 c.4096+1G>A variant. It has been obtained using the UPGMA clustering method through DendroUPGMA online software. The numbers on each branches indicate the number of generations.

“The founder mutation model” implies that a given chromosome carrying the c.4096+1G>A pathogenic variant replicated at some point, giving rise to two independent lineages (from which other branches later arose). The segment of identical haplotype was gradually shortened by random recombination events on both sides of the gene, leaving in the present descendants a shared haplotype whose expected length is a function of the number of generations elapsed since the original replication (Figure 3). We estimated this function using computer simulations of the recombination process in the particular chromosomal segment carrying the variant.

The distance square matrix in number of generations was used to generate a dendrogram using an unweighted pair group method with arithmetic mean (UPGMA) clustering (Figure 3). The obtained dendrogram, or extended family tree, shows that individual P019 is the most distant from all the other carriers. In fact, P019 shares a very short haplotype segment with them, about 0.9 cM on average. This length allows the existence of MRCA for all individuals in the sample. The MRCA would have lived about 155 generations ago.

**Figure 4:**
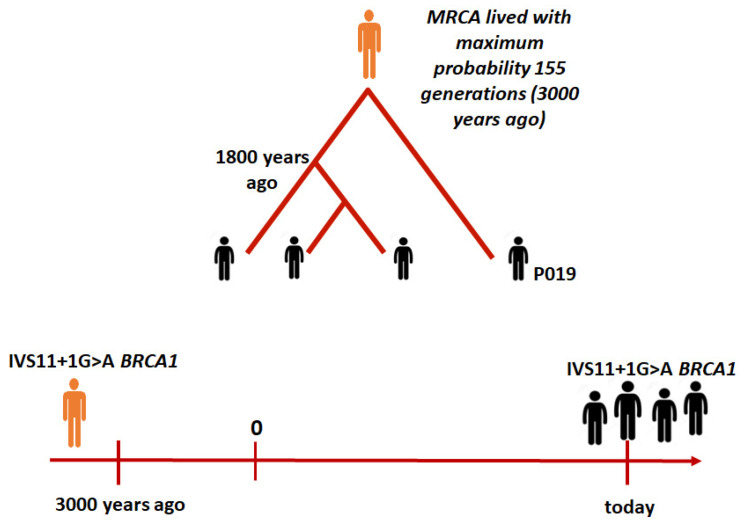
The timeline shows the temporal distance separating today’s variant carriers of c.4096+1G>A from their ancestor, who lived approximately 3000 years ago, around 155 generations back.

Given that a generation is about 20–25 years, this means that the common ancestor of all individuals in the sample carrying the variant in question lived more than 3000 years ago. However, most of the index cases refer to a recombination event in a common ancestor who lived 1800 years ago (Figure 4).

After repeating the analysis using the method of Gandolfo et al. [27], the age of the variant was estimated to be 2425 years, assuming again 25 years per generation. Nevertheless, the confidence limits of the estimation (1800–3250) included the 3000-year value obtained using the method of Marroni et al. [18]. For interpreting the results using the Gandolfo method, we considered a correlated genealogy, since subsets of the sampled individuals have common ancestry earlier than the MRCA for the entire sample.

## 3. Discussion

In our study, we used Marroni’s phylogenetic method with some modifications to estimate the time to MRCA of the *BRCA1* c.4096+1G>A founder pathogenic variant. First, a genomic region around *BRCA1* was defined by genetic typing of the collected families for 4130 SNPs. The next step was to determine the haplotype lengths shared by all the pairs of variant carriers and to enter them into a triangular similarity matrix. This matrix was then converted into a distance matrix needed to infer the evolutionary tree of the c.4096+1G>A variant. Utilizing the phylogenetic approach presents numerous benefits as demonstrated in multiple studies, including the investigation conducted by Marroni et al. [18] on the *BRCA1* 1499insA founder mutation. A crucial advantage is the ability to reconstruct the evolutionary history of a founder variant by drawing a dendrogram of the variant carriers. Time-dependent branches of the dendrogram are representative of the family trees of all variant-carrying haplotypes.

Due to its upsides, this method has been extensively used in literature. It was exploited by Tuazon et al. [36] to characterize the haplotype flanking the *BRCA1* c.3331_3334delCAAG pathogenic variant in six different populations (Colombia, Chile, Portugal, Angola, Brazil, and Spain). They demonstrated the common Iberian origin of the pathogenic variant by tracing it back to between 1960 and 2400 years ago. Ndiaye et al. [37] studied the *BRCA1* c.815_824dupAGCCATGTGG pathogenic variant identified in 15 index cases in Senegal. Using seven microsatellites flanking the BRCA1 pathogenetic variant, they identified a common 400KB haplotype. The pathogenetic variant originated in Senegal about 1400 years ago, and probably spread through the slave trade.

Using the method of Gandolfo [35], Shaw et al. [38] identified an ancient Chinese *BRCA1* founder variant dating back 77.9 generations, possibly common among individuals of southern Han Chinese descent. They found a 590KB haplotype block that was conserved in those carrying the variant and absent in those who did not carry the variant. The list of articles that confirm our results using the same approach is much longer [39,40].

In our work, using the haplotype sharing method, we confirmed the hypothesis of a founder effect for this variant in the Tuscany region. Indeed, all the individuals analyzed share a minimum common haplotype around the *BRCA1* gene. The analysis also showed that P019 was the most distant individual from all other carriers. He shares a very short haplotype segment with them, on average 0.9 cM in size. This length suggests that the most recent common ancestor lived about 155 generations ago—3000 years ago.

However, with the exception of P019, all individuals share a most recent common ancestor dating back approximately 1800 years, suggesting a recent arrival of P019’s ancestor in the Tuscany region. The findings were corroborated using the Gandolfo method [35], estimating the age of the genetic variant at 2425 years. This aligns with the previous method, which suggested an age of 3000 years, falling within the upper confidence limit of 3250 years. An alternative explanation is that the P019 haplotype’s similarity may be due to identity by state than by descent. This would imply that the mutation leading to the c.4096+1G>A variant occurred independently twice, affecting the same haplotype block. While this possibility cannot be ruled out, its likelihood is considerably lower than that of a shared ancestry.

The distinct haplotype configuration of P019 indicates multiple migration events. Approximately 1800 years ago, a wave of migration introduced this genetic variant *BRCA1* c.4096+1G>A to Tuscany, from where it was subsequently disseminated. Additionally, a more recent migration occurred, bringing the ancestors of individual P019 to Tuscany. It is therefore assumed that the ancestor of individual P019 belongs to another group of carriers, descended from the most recent common ancestor, who lived earlier in time and was geographically distant compared to the Tuscan group.

Tuscany, indeed, has been affected by various waves of migration since the year 1000, although they have not been demographically significant. However, many people from Italy and other European countries arrived in different parts of the region. They were mainly motivated by trade, work, and study. From 1472 for example, the Tuscan city of Pisa received French, German, and Spanish students, as well as students from other parts of Italy [41]. This would explain why individual P019, apparently geographically close to all the others, is so distant from them genealogically. It would certainly be useful to have more information on the geographical origin of the P019 family to better clarify the reason for the different haplotype.

Knowing whether a particular pathogenic variant is common in a population can be very useful in planning effective screening and prevention strategies. This is particularly true in developing countries, where resources for genetic analysis are limited.

## 4. Materials and Methods

### 4.1. Families and Genotyping

#### 4.1.1. Description of Recruited Families

Among 3803 probands referred to our center for BRCA1 mutation analysis, 27 apparently unrelated subjects were carriers of the c.4096+1G>A variant. Upon recruiting their at-risk relatives for genetic counseling, 27 additional subjects were typed (11 carriers), for 54 total available DNA samples. As the number of allotted slots in the genome-wide typing platform was 48, the six least informative subjects were excluded. In the end, the study’s final sample included 15 singleton, 5 doubleton, 5 tripleton, and 2 quadrupleton families. The most remote carrier progenitor of each family was selected for haplotype analysis (27 subjects). The clinical-pathological characteristics of recruited individuals are reported in Table 1.

#### 4.1.2. DNA Extraction and Genotyping

DNA was extracted from peripheral blood lymphocytes using a QIAsymphony apparatus (Qiagen, Hilden, Germany). DNA concentration and quality were then assessed and verified using a NanoDropTM 2000 spectrophotometer (Thermo Fisher Scientific, Inc., Waltham, MA, USA).

All subjects were typed for 4130 SNPs (21 within *BRCA1*) localized in a region of approximately 20 Mb of chr-17 (24.6 cM), between rs425809 and rs203076. An Illumina Infinium Oncoarray (Illumina, San Diego, CA, USA) containing 533,531 cancer-associated SNPs distributed across the genome was used to generate the data [42].

### 4.2. Haplotype Reconstruction

#### 4.2.1. Determination of the Gametic Phase of the Markers

The gametic phase of the markers included in the region of interest was inferred using the SHAPEIT software [43], which requires their genetic map. The HapMap Phase II b37 database (http://hapmap.ncbi.nlm.nih.gov/downloads/recombination/2011-01_phaseII_B37; accessed on 1 October 2023) was used for this purpose. Finally, the output file generated by SHAPEIT was visually checked in order to fix possible inaccuracies performed by the software.

The haplotype length (in base pairs, bp) shared by pairs of c.4096+1 G>A variant carriers, derived from the difference in genomic position of markers at the 5’ and 3’ ends, was converted to centiMorgan (cM) using the HapMap Phase II b37 database. Using the haplotype length in cM, shared by pairs of variant carriers, a quadratic symmetric similarity matrix was generated.

#### 4.2.2. Inferring the Genealogy of the c.4096+1G>A Variant

Following Marroni’s phylogenetic method [18], the similarity matrix of segment length (cM) shared by all pairs of variant carriers was converted by computer simulation into the number of generations separating each pair. This number was then doubled to express the amount of meiosis. The resulting distance matrix was subjected to UPGMA [44] clustering, a simple hierarchical clustering method that generates dendrograms by successive steps starting from a distance matrix (Figure 3). For this purpose, the online software DendroUPGMA was used (http://genomes.urv.cat/UPGMA/, accessed on 1 October 2023).

The lengths of the shared haplotype segments were determined following the methodology established in a prior investigation conducted by Marroni et al. [18]. Specifically, these segment lengths were computed as the cumulative distance between the last markers on both sides of the *BRCA1* variant, exhibiting identical alleles among all variant carriers.

Additionally, to confirm the consistency of the results produced by Marroni’s algorithm, we applied a different method for variant dating in small populations. Specifically, the Gandolfo method [35] was applied via the online application available at https://shiny.wehi.edu.au/rafehi.h/mutation-dating/ (accessed on 1 October 2023). The data provided to the online software were processed using a custom-made Python script.

## Figures and Tables

**Table 1:** Clinical-pathological characteristics of recruited individuals. Individuals of the same family are enclosed by borders. Probands are shown in bold.

ID	Gender	Age of the Last Follow-Up or Age of Death	Relationship to the Probamd	c.4096+1G>A *BRCA1*	Tumor	Age of Onset
**P011**	**F**	**80**	Proband	***Carrier***	**HGSOC**	**52**
**P012**	**F**	**76**	Proband	***Carrier***	**Breast/HGSOC**	**61/73**
**P013**	**F**	**77**	Proband	***Carrier***	**Breast/HGSOC**	**62/67**
P013a	M	75	Brother	*Wild-type*	Healthy	
**P014**	**F**	**51**	Proband	***Carrier***	**Breast**	**49**
**P015**	**F**	**49**	Proband	***Carrier***	**Breast**	**39**
**P016**	**F**	**59**	Proband	***Carrier***	**Breast**	**49**
P016a	F	62	Sister	*Carrier*	Healthy	
P016b	F	26	Nephew	*Carrier*	Healthy	
P016c	F	80	Mother	*Wild-type*	Breast	79
**P017**	**F**	**33**	Proband	***Carrier***	**Breast**	**25**
P017a	M	59	Father	*Carrier*	Healthy	
P017b	F	56	Mother	*Wild-type*	Breast	47
**P018**	**F**	**76**	Proband	***Carrier***	**Breast**	**60**
P018a	F	56	Nephew	*Wild-type*	Healthy	
P018b	F	90	Sister	*Carrier*	Breast	86
**P019**	**F**	**43**	Proband	***Carrier***	**Breast**	**37**
**P0110**	**F**	**63**	Proband	***Carrier***	**Breast**	**50**
P0110a	F	38	Daughter	*Wild-type*	Healthy	
**P0111**	**F**	**89**	Proband	***Carrier***	**Breast**	**72**
P0111a	F	65	Daughter	*Carrier*	Healthy	
P0111b	M	33	Nephew	*Carrier*	Healthy	
P0111c	F	28	Nephew	*Wild-type*	Healthy	
**P0112**	**F**	**44**	Proband	***Carrier***	**Breast**	**32**
P0112a	M	71	Father	*Wild-type*	Healthy	
P0112b	F	71	Mother	*Carrier*	Healthy	
**P0113**	**F**	**39**	Proband	*Carrier*	Healthy	
**P0114**	**F**	**54**	Proband	***Carrier***	**Breast**	**49**
**P0115**	**F**	**69**	Proband	***Carrier***	**Breast/Uterus**	**55/65**
P0115a	F	44	Daughter	*Carrier*	Healthy	
**P0116**	**F**	**79**	Proband	***Carrier***	**Breast**	**48**
P0116a	F	58	Daughter	*Carrier*	Healthy	
P0116b	F	49	Daughter	*Wild-type*	Healthy	
**P0117**	**F**	**63**	Proband	***Carrier***	**HGSOC**	**59**
**P0118**	**F**	**81**	Proband	***Carrier***	**Breast/HGSOC**	**77/72**
P0118a	F	56	Daughter	*Wild-type*	Healthy	
**P0119**	**F**	**74**	Proband	***Carrier***	**HGSOC**	**61**
**P0120**	**F**	**65**	Proband	***Carrier***	**HGSOC**	**62**
**P0121**	**F**	**58**	Proband	***Carrier***	**Breast**	**54**
**P0122**	**F**	**46**	Proband	***Carrier***	**Breast**	**44**
P0122a	M	75	Father	*Wild-type*	Healthy	
P0122b	F	75	Mother	*Carrier*	Healthy	
**P0123**	**F**	**83**	Proband	***Carrier***	**HGSOC**	**78**
**P0124**	**F**	**54**	Proband	***Carrier***	**Breast**	**52**
**P0125**	**F**	**81**	Proband	***Carrier***	**HGSOC**	**78**
**P0126**	**F**	**66**	Proband	***Carrier***	**HGSOC**	**58**
P0126a	F	41	Daughter	***Carrier***	Healthy	
**P0127**	**F**	**47**	Proband	***Carrier***	**Breast**	**45**

## Data Availability

The data generated during the study are available upon request.
